# Genetic Variant Overlap Analysis Identifies Established and Putative Genes Involved in Pulmonary Fibrosis

**DOI:** 10.3390/ijms24032790

**Published:** 2023-02-01

**Authors:** Karlijn Groen, Joanne J. van der Vis, Aernoud A. van Batenburg, Karin M. Kazemier, Jan C. Grutters, Coline H. M. van Moorsel

**Affiliations:** 1 Department of Pulmonology, St. Antonius ILD Center of Excellence, St. Antonius Hospital, 3435 CM Nieuwegein, The Netherlands; 2 Department of Clinical Chemistry, St. Antonius ILD Center of Excellence, St. Antonius Hospital, 3435 CM Nieuwegein, The Netherlands; 3 Center of Translational Immunology, University Medical Center Utrecht, 3508 GA Utrecht, The Netherlands; 4 Division of Hearts and Lungs, University Medical Center Utrecht, 3584 CX Utrecht, The Netherlands

**Keywords:** idiopathic pulmonary fibrosis, genetics, telomeres, *TDP1*, *TOM1L2*

## Abstract

In only around 40% of families with pulmonary fibrosis (PF) a suspected genetic cause can be found. Genetic overlap analysis of Whole Exome Sequencing (WES) data may be a powerful tool to discover new shared variants in novel genes for PF. As a proof of principle, we first selected unrelated PF patients for whom a genetic variant was detected (n = 125) in established PF genes and searched for overlapping variants. Second, we performed WES (n = 149) and identified novel potentially deleterious variants shared by at least two unrelated PF patients. These variants were genotyped in validation cohorts (n = 2748). In 125 unrelated patients, a potentially deleterious variant was detected in known PF genes of which 15 variants in six genes overlapped, involving 51 patients. Overlap analysis of WES data identified two novel variants of interest: *TOM1L2* c.421T > C p.(Y141H) and *TDP1*c.1373dupG p.(S459fs*5), neither gene had been related to pulmonary fibrosis before. Both proteins were present in the alveolar epithelium. No apparent characteristics of telomere disease were observed. This study underlines the potential of searching for overlapping rare potentially deleterious variants to identify disease-associated variants and genes. A previously unreported variant was found in two putative new PF genes, but further research is needed to determine causality.

## 1. Introduction

Pulmonary fibrosis (PF) in the context of interstitial lung disease is characterized by progressive scarring of the lung and reduced survival. The most severe form of pulmonary fibrosis is idiopathic pulmonary fibrosis (IPF), a progressive disease of unknown cause with a median survival of 3–5 years after diagnosis [1]. Genetics is known to play an important role in disease development. Approximately 20% of IPF patients have familial pulmonary fibrosis (FPF) with disease usually being transmitted in an autosomal dominant way. A genetic cause can also be suspected in sporadic cases, based on early age of onset or disease phenotype. An increasing number of PF genes is known and the vast majority are related to telomere biology. In approximately 35% of cases, mutations are found in telomere-related genes—such as *TERC*, *RTEL1*, and *PARN*, but most commonly the *TERT* gene [2,3]. Patients with telomere related gene mutations frequently show short telomere length in blood and lung biopsy, increased DNA-damage in lung tissue, and clinical signs and symptoms of short telomere syndromes [3,4,5,6]. In only 3–8% of suspected genetic PF cases, a mutation can be found in genes of the surfactant system [2,3].

The phenotype of mutation carriers usually involves PF, including IPF, but also other forms of pulmonary fibrosis, such as fibrotic hypersensitivity pneumonitis (fHP) and non-classifiable interstitial lung disease (ILD). However, other lung diseases—such as pediatric ILD, neonatal RDS, and lung cancer in case of surfactant mutations—or pulmonary arteriovenous malformations and emphysema in case of telomere related mutations have also been observed, as reviewed by Hoffman et al. [7] and Van Moorsel et al. [2]. An increasing number of genes, here referred to as PF genes, are now known to be involved in genetic PF. Nonetheless, a causal variant—often called a mutation—can only be found in around 40% of the cases with a suspected monogenetic origin [2,3].

Several strategies for discovering new PF genes and variants involved in PF can be applied. Candidate gene approaches yielded important results such as for *SFTPC* [8], *TERT*, and *TERC* [9]. Linkage analysis yielded *SFTPA2* [10] and analysis of genome wide data yielded *RTEL1* through segregation analysis [11]. However, this approach requires DNA from multiple affected family members, something that is not often available in this rare disease that mostly presents in the sixth or seventh decade of life and with such short survival time. Alternatively, an genetic overlap based strategy can be applied in unrelated patients with similar disease phenotypes, whereby variants in a single, thus overlapping, gene are sought [12]. As an example, analysis of genome wide data yielded *PARN* through gene burden analysis [13]. Interestingly, several identical variants have been observed to cause PF in multiple unrelated patients, such as the *SFTPC* p.I73T and *TERT* p.R669W mutations [14,15,16,17,18,19]. We thus hypothesized that a modified overlap based strategy, namely genetic variant overlap analysis, may be used to discover new variants in novel genes for PF. Using this strategy, we here search for identical, overlapping variants in unrelated patients with PF.

The St. Antonius ILD Center of Excellence manages the Dutch national ILD-biobank, gathering blood samples and data from patients with ILD in this small-sized country. First, we performed a proof of principle analysis for the genetic variant overlap analysis. To this end, we retrospectively summarized the deleterious variants in known PF genes that had been observed in unrelated patients with PF in the Biobank. Then we identified the number of variants that were shared, thus overlapping, between the patients. Second, we applied the overlap-based strategy as an explorative approach to identify new variants and genes that may be causal to PF. We therefore performed whole exome sequencing (WES) and searched for novel, deleterious variants that are shared amongst multiple unrelated patients. Discovered novel variants were genotyped in ILD-biobank samples, and then analyzed for expression in lung tissue and characteristics of telomere related disease.

## 2. Results

### 2.1. Overlap of Variants in Established PF Genes

**Figure 1:**
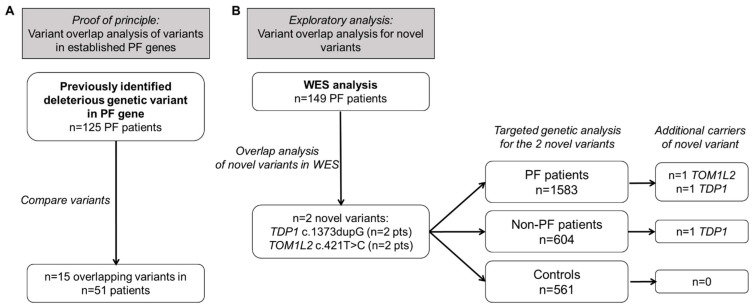
Variant overlap analysis in unrelated Dutch patients with pulmonary fibrosis (PF). (**A**) proof of principle for variant overlap analysis showing the number of PF patients from the ILD Biobank with a previously identified deleterious variant in a known PF gene. Deleterious variants had been classified as pathogenic, likely pathogenic, or a variant of uncertain significance (VUS). (**B**) PF cohort for identification of novel PF genes by variant overlap analysis and screening of a cohort of PF and non-PF patients and controls for these novel variants. PF = pulmonary fibrosis.

**Figure 2:**
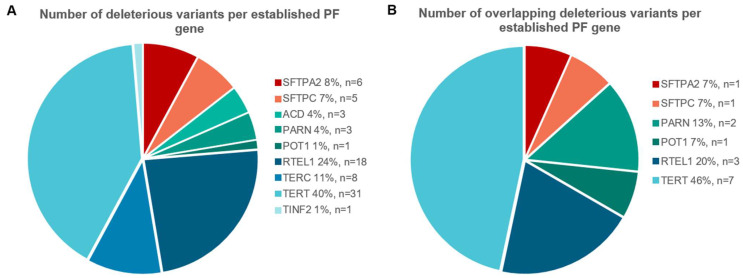
(**A**) Distribution of number of different variants per established dominant PF gene. A total of 65 out of 76 variants are observed in telomere related genes (blue/green), accounting for 85% of the variants in total. Eleven variants are located in surfactant related genes (red/orange); (**B**) Distribution of number of overlapping variants per established PF gene. Thirteen out of 15 variants involve telomere related genes, accounting for in total 87% of the observed overlap.

To determine if searching for overlapping variants can be a useful strategy, we checked whether overlapping variants are present amongst our cohort of PF patients with a previously identified deleterious variant. Genetic variants classified by clinical genetic criteria as variants of unknown significance (VUS), likely pathogenic (LP), or pathogenic (P) variant in a PF gene had been previously identified in 125 patients (Figure 1A). These 125 patients jointly carried variants in nine different genes: two surfactant related genes (*SFTPA2*, *SFTPC*) and seven telomere related genes, of which the majority were present in the *TERT* gene. Figure 2A shows the number of different dominant variants per PF gene (variants located in genes associated with recessive disease were omitted here). Fifteen variants were found in more than one presumably unrelated patient (Table 1). The distribution of these shared variants per gene are shown in Figure 2B. Overlap was detected in two surfactant genes and four out of seven telomere related genes and was most common in *TERT* (7 variants in 28 patients; Figure 2B). No overlap was present in *ACD*, *TERC*, or *TINF2*. Genealogical research to determine possible relatedness, identified distant relatedness for 4 out of 13 patients carrying the *TERT* p.(Arg669Trp) variant [19], 3 out of 4 patients carrying the *TERT* p.(Pro771Leu) variant, and 2 out of 2 patients with the *POT1* p.(Leu259Ser) [20], *RTEL1* p.(Leu658del) or *TERT* p.(Tyr576His) variant. Thus, there is significant overlap of rare deleterious variants in established PF genes.

### 2.2. Variant Overlap Analysis to Identify Novel Variants in PF

**Figure 3:**
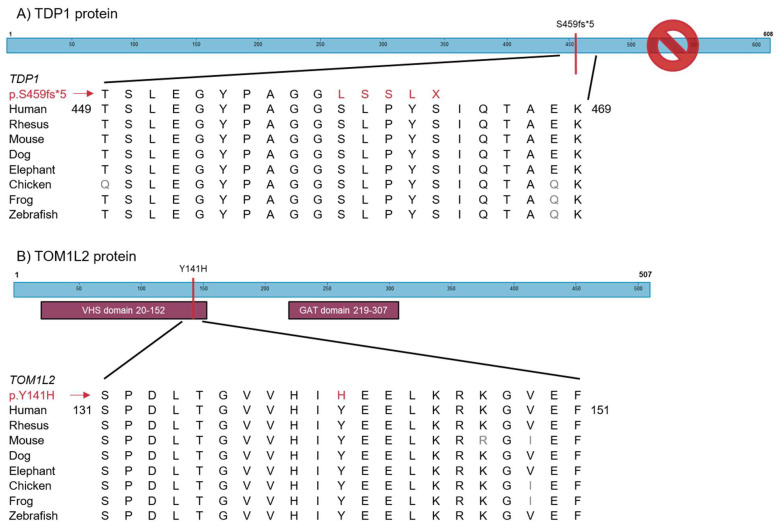
(**A**) Schematic representation of the TDP1 protein and amino acid conservation around the c.1373dupG p.(S459fs*5) variant. The variant is predicted to cause a frameshift resulting in a premature stop codon (X); (**B**) Schematic representation of the TOM1L2 protein with its two functional domains. The predicted p.(Y141H) amino acid alteration caused by the c.421T > C variant is located in the VHS domain. Amino acid conservation around novel variants in TDP1 and TOM1L2 across eight species with the conserved residues in black and the new variants in red.

Genetic variant overlap analysis was then applied to identify novel variants and genes involved in PF. To this end, WES was performed on blood derived DNA in 149 unrelated PF patients. WES data were subjected to filtering to select variants that were: (1) predicted deleterious by in silico prediction models; (2) were absent from population databases; and (3) were shared between at least two patients. After this variant filtering, 25 variants remained, of which 22 were excluded after visual inspection of the raw data. Visual inspection of the *TBXT* c.457A > C variant was inconclusive, but the variant was absent from subsequent Sanger sequences, and was thus considered a sequencing artefact in the WES. Two variants of interest remained, namely *TOM1L2* c.421T > C and *TDP1* c.1373dupG (Table 2). Both were present in two patients in the WES cohort. The target of myb1-like 2 membrane trafficking protein *(TOM1L2)* c.421T > C missense variant is predicted to cause a substitution of the highly conserved hydrophobic tyrosine residue with a positively charged histidine (p.(Y141H)) and is located in the VHS domain (Figure 3A). The tyrosyl-DNA phosphodiesterase 1 (TDP1) c.1373dupG p.(S459fs*5) variant is predicted to cause a frameshift introducing a premature stop codon likely leading to nonsense mediated decay. We examined the WES data for other rare variants (frequency < 1% in population databases) in these two genes. Two more variants were identified in *TDP1*. One variant was found in two unrelated individuals (*TDP1* c.1443G > A). This variant did not pass the original filtering criteria due to its presence in population databases. However, available WES data from a brother with pulmonary fibrosis of one of these patients indicated lack of segregation. The other *TDP1* variant (c.1580delA (p.K527fs*6); rs770659676) is a very rare variant which we found in one patient with FPF. However, no material was available for segregation analysis, and this variant was therefore not investigated further. To identify additional carriers of these variants, targeted genotyping was applied in an extended cohort of pulmonary fibrosis patients (n = 1583). We identified one additional heterozygous carrier of the *TOM1L2* variant and one heterozygous carrier of the *TDP1* c.1373dupG variant in this cohort. Targeted genotyping in non-PF patients (n = 604) identified one non-PF patient carrying the *TDP1* c.1373dupG variant. Neither variant was identified in healthy controls (n = 561).

### 2.3. Novel Variant Patient Characteristics

The *TOM1L2* c.421T > C variant was present in three PF patients each with a different diagnosis, namely fHP, IPF, and nonclassifiable interstitial pneumonia (NCIP) (see Table 3). Patient 1 was a 52-year-old male diagnosed with fHP. A causative antigen could not be identified. The patient’s mother had been diagnosed at age 77 with pulmonary fibrosis. On HRCT a pattern of probable usual interstitial pneumonia (UIP) was detected with subpleural reticulation, traction bronchiectasis with basal and peripheral predominance and absence of honeycombing. He received a bilateral lung transplant at age 60. The second patient was a 47-year-old male diagnosed with sporadic IPF and WES was performed as part of an earlier study [4]. Histologic analysis showed a UIP pattern with concomitant DIP-like features. He underwent a unilateral lung transplant at 51 years old. The third patient was a 55-year-old male diagnosed with NCIP with a suspicion of PF. An HRCT pattern of NSIP and a histological pattern of UIP with lymphoid hyperplasia containing B lymphocytes and endogenous lipoid pneumonia were observed.

*TDP1* c.1373dupG was initially found in two IPF patients (Patients 4 and 5). Screening in a broader cohort of PF patients, non-PF patients and healthy controls identified two more heterozygous carriers: one patient with lymphangioleiomyomatosis (LAM) (patient 6) and one patient with COPD and PF (patient 7). Patient 4 is a male patient with a familial background of lung fibrosis who was diagnosed with IPF at age 62. The HRCT shows a UIP pattern with suspected diffuse alveolar damage (DAD) and includes emphysematous changes. In a diagnostic biopsy, a UIP pattern with DAD was observed. Patient 5 is a sporadic male IPF patient diagnosed at age 50. Both radiology and histology showed a UIP pattern, which is accompanied by paraseptal emphysema on HRCT. He received a bilateral lung transplant at age 52. Patient 6 is a 46-year-old female diagnosed with sporadic LAM. LAM diagnosis was based on presence of bilateral angiomyolipomas in the kidneys and a strongly increased serum VEGF-D level. Additionally, HRCT displayed mild small thin-walled cysts in the lung. Patient 7 was a 56-year-old male diagnosed with COPD GOLD IV with an HRCT with extensive emphysema and bronchiolitis. He therefore received a unilateral lung transplant (left) at age 57. After lung transplantation, the patient developed progressive fibrosis with radiological UIP pattern features in the native right lung. Analysis of explant lung tissue showed signs of fibrosis i.e., accumulation of interstitial fibroblast in addition to emphysema.

### 2.4. TOM1L2 Presence in Lung Cells

**Figure 4:**
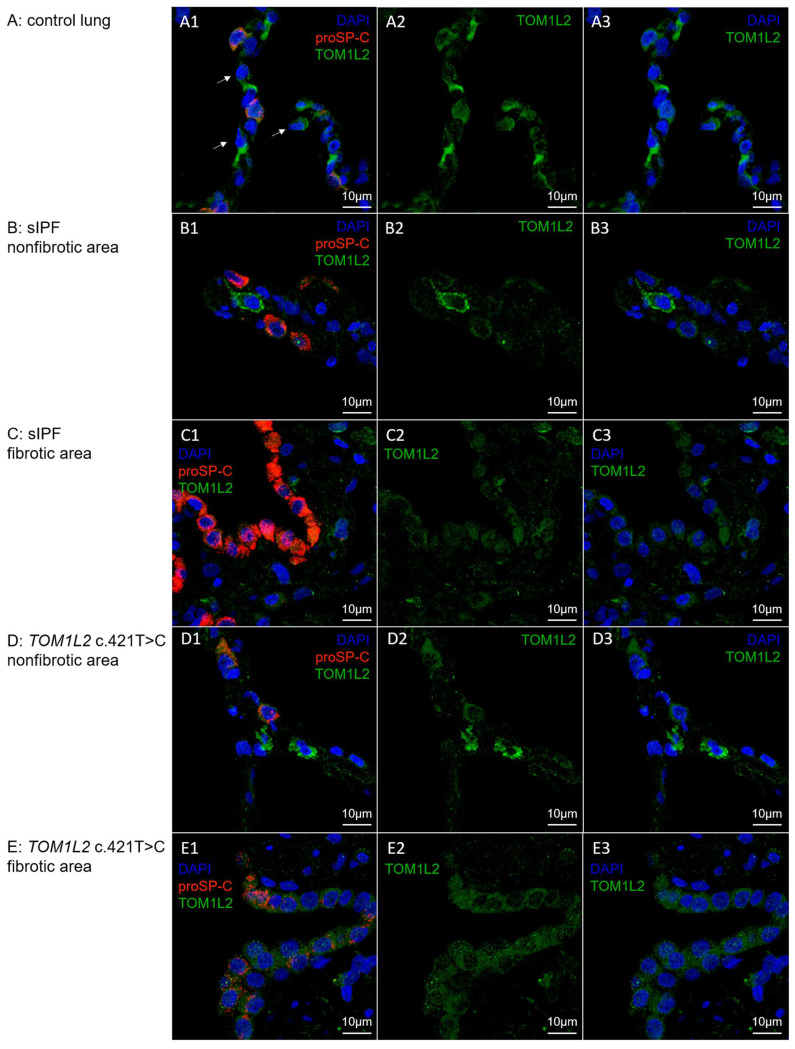
TOM1L2 protein expression in alveoli of (**A**) control tissue; (**B**,**C**) non-fibrotic and fibrotic lung area of a sporadic IPF patient, respectively; and (**D**,**E**) non-fibrotic and fibrotic lung area of a TOM1L2 c.421T > C variant carrier (Patient 3), respectively. (**A1**–**E1**): merge of mouse- antiTOM1L2-selective immunofluorescence staining (green), proSP-C specific staining as a marker for alveolar type 2 cells (red) and DAPI DNA staining (blue); (**A2**–**E2**): only TOM1L2 specific staining; (**A3**–**E3**): TOM1L2 staining merged with DAPI staining. Arrows indicate proSP-C negative cells in the alveoli that are positive for TOM1L2 staining.

Double staining in lung tissue of control subjects showed that TOM1L2 protein is located in Type 2 alveolar epithelial cells (AT2 cells) as well as in proSP-C negative cells (indicated by the arrows) in the alveoli of control lung (Figure 4A). Localization is predominantly cytosolic. As a confirmation for the mouse-anti-TOM1L2 antibody, the staining was repeated with a rabbit-anti-TOM1L2 antibody on subsequent slides from the same sample which showed a similar pattern (see Supplemental Results S3). In a sporadic IPF patient and a PF *TOM1L2* c.421T > C carrier, TOM1L2 positive staining was present in non-fibrotic (B,D) and fibrotic (C,E) lung tissue in (hyperplasic) AT2 cells, but also in unspecified proSP-C negative cells.

**Figure 5:**
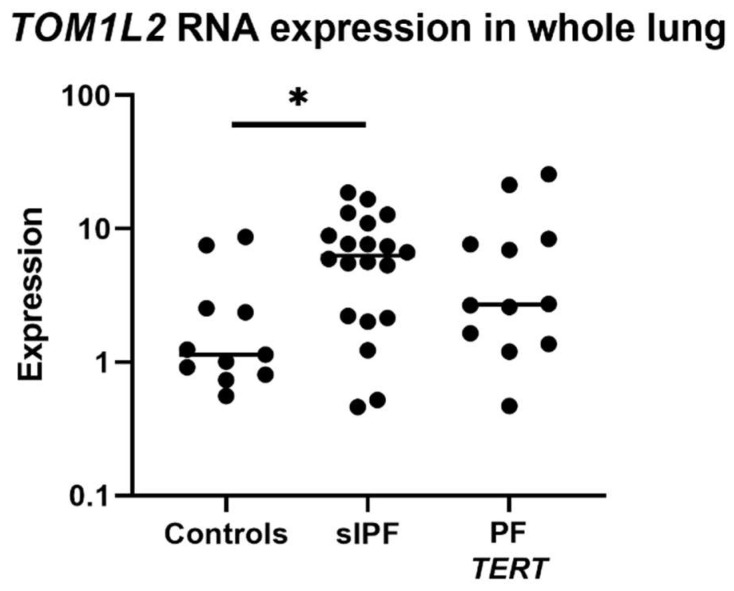
*TOM1L2* RNA expression in whole lung tissue from controls, sporadic IPF patients (sIPF) and IPF patients with a *TERT* mutation (TERT). *TOM1L2* expression is relative compared to the mean of three reference genes multiplied by 1000. * = *p* < 0.05.

*TOM1L2* RNA expression was measured in whole lung tissue from controls, sporadic IPF, and *TERT* mutation carriers. Low expression of reference genes prevented analysis in *TOM1L2* variant carriers. *TOM1L2* RNA expression was significantly increased in sporadic IPF (sIPF) compared to control lung (*p* = 0.040) (Figure 5). When sIPF and TERT mutations carriers were grouped, expression was significantly higher in this group as compared to controls (*p* = 0.012; see Supplemental Results S4).

### 2.5. TDP1 Expression in the Lung

**Figure 6:**
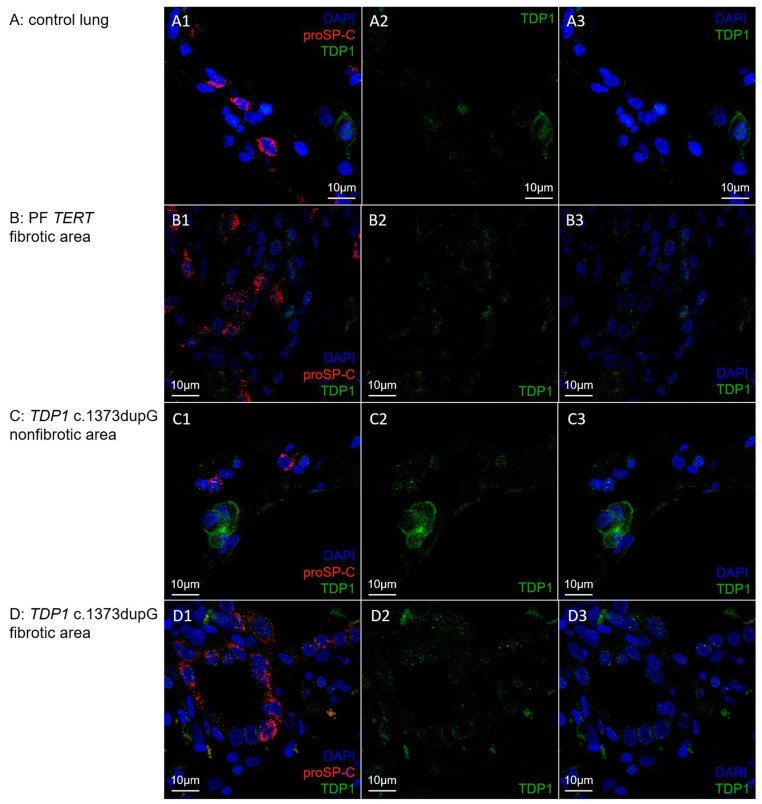
TDP1 protein expression in control lung (**A**), fibrotic area of PF TERT lung (**B**), and non-fibrotic (**C**) and fibrotic (**D**) lung area of TDP1 c.1373dupG variant carrier (Patient 5). (**A1**–**D1**): merge of TDP1-selective immunofluorescence staining (green), proSP-C specific staining as a marker for type 2 alveolar cells (red) and DAPI DNA staining (blue); (**A2**–**D2**): only TDP1 staining; (**A3**–**D3**): overlay of TDP1 staining with DAPI staining.

TDP1 is present in cells in the alveoli of control lung, as well as in lung tissue of a *TERT* mutation carrier and Patient 5 carrying the *TDP1* c.1373dupG variant (Figure 6). Staining is of limited intensity and most pronounced in the cytosol of proSP-C negative cells, but it can also be observed in the nucleus of both proSP-C positive and negative cells. No differences in TDP1 intensity were optically detected between carriers and non-carriers of the *TDP1* c.1373dupG variant.

### 2.6. Telomere Length

**Figure 7:**
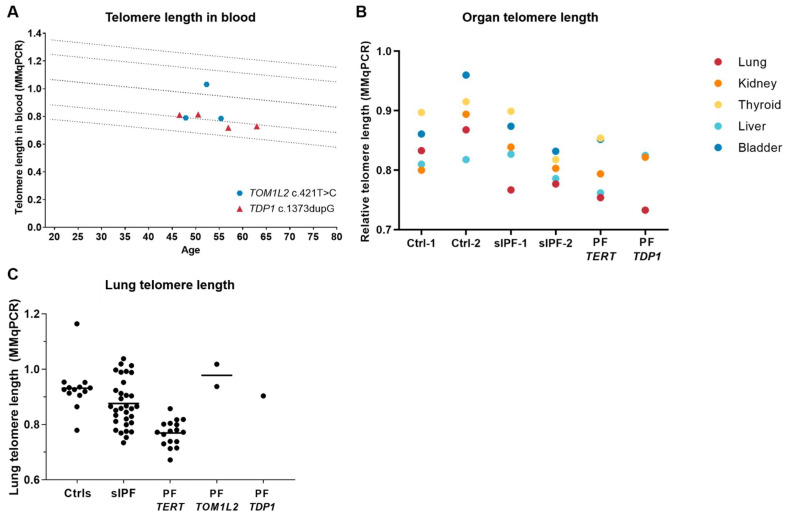
(**A**) Telomere length in blood plotted against age as measured by MMqPCR. Circles indicate patients carrying the *TOM1L2* c.421T > C variant, triangles indicate patients carrying the *TDP1* c.1373dupG variant. The straight line indicates 50th percentile of control subjects; dotted lines the 1st, 10th, 90th, and 99th percentile of control subjects. (**B**) Telomere length in autopsy FFPE tissue from different organs. PF *TERT* = PF patient with a mutation in the *TERT* gene; PF *TDP1* = Patient 4; (**C**) Telomere length in biopsy tissue of whole lung. Data on controls, IPF, and *TERT* mutation carriers in (**B**,**C**) were previously published [4]. No statistical analysis was applied due to small group size.

As PF causing mutations are mostly located in telomere related genes and associated with short telomeres, we sought whether variant carriers showed signs of short telomeres. Telomere length in blood of variant carriers is close to the 10th percentile for age except for patient 1 with *TOM1L2* c.421T > C variant who had telomere length above the 50th percentile (Figure 7A). Telomere length in lung and other organs based on T/S ratio was determined in autopsy tissue of *TDP1* variant carrier Patient 4 (Figure 7B). In IPF and *TERT* PF tissues, telomere length is shortest in the lungs, which is also observed in the *TDP1* variant tissue. Telomere length in lung biopsy is comparable to controls (Figure 7C).

### 2.7. Telomere Length and DNA Damage in AT2 Cells of TOM1L2 and TDP1 Variant Carriers

**Figure 8:**
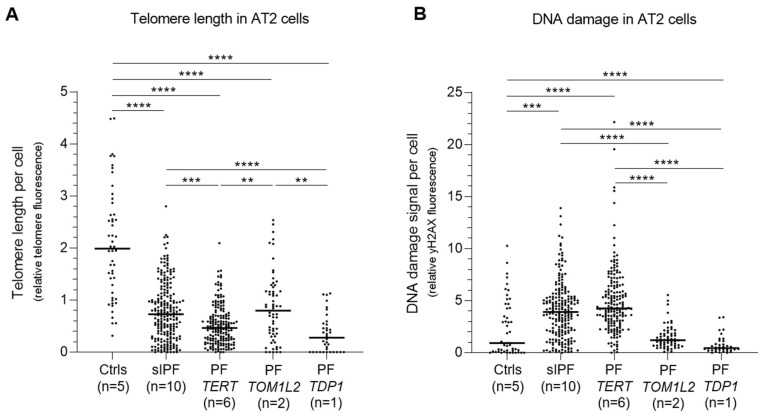
Cell specific quantification of telomere length **(A)** and DNA damage **(B)** in alveolar type 2 (AT2) cells of control, sporadic IPF (sIPF) and pulmonary fibrosis with a mutation in *TERT* or variant in *TOM1L2* or *TDP1*. Telomere length is measured by FISH and DNA damage by immunofluorescence for γH2AX. **: *p* < 0.01; ***: *p* < 0.001; ****: *p* < 0.0001, calculated by Kruskal–Wallis multiple comparison tests and Dunn’s multiple comparison test.

AT2 cell telomere length of *TOM1L2* and *TDP1* variant carriers (Patients 2, 3, and 5) is significantly shorter than in controls. AT2 cell TL in *TOM1L2* variant carriers is comparable to that of sporadic IPF patients, whereas that of the *TDP1* variant carrier (Patient 5) is significantly shorter (Figure 8). DNA damage in AT2 cells of both variants is considerably lower than that of both sporadic and *TERT* PF patients.

## 3. Discussion

In this study, we show that there is considerable overlap of the genetic variants in established PF genes that were present in unrelated patients. Additionally, a strict search for putative damaging variants shared between unrelated patients revealed two variants in two novel genes that may be involved in PF.

Genetic variant overlap may be caused by shared ancestral origin, or by recurrent de novo mutations. Among the well-known mutations in PF, both can be found. The *SFTPC* mutation I73T has been discovered in patients all over the world, inherited or de novo [15,16,18](. It is the most common of all surfactant mutations, and was previously shown to have originated on different haplotypes in affected children and in adults, proof of recurrent mutation at a mutational hotspot [18,28]. We detected a particularly high number of overlapping variants in telomere related genes, particularly in *TERT*. Previously, the *TERT* c.2371G > A and c.2599G > A in *cis* [35] and the *TERT* c.2005C > T mutation that was also described here [19] have each been reported to share a common ancestor. For multiple telomere related variants, post hoc genealogical research resulted in the detection of distant relatedness of carriers of identical mutations. The distribution of these variants may be the result of reduced penetrance or genetic anticipation. In families with TRG mutations, telomere length is passed on from generation to generation; hence, in mutation carriers, telomeres shorten in successive generations. Therefore, inheritance through several generations may be required before a telomere related mutation may have caused significant telomere shortening necessary for disease manifestation [19]. Comparison of surrounding haplotypes can confirm distant relatedness among reported carriers, though this was not performed here. Given the considerable overlap in our cohort but also in patients found worldwide, a general database containing detected variants in pulmonary disease, may help establish the pathogenicity of observed variants. Most importantly, the significant overlap of variants in established PF genes showed that such an analysis could also be a powerful tool to discover novel variants in novel genes.

Overlap analysis in our WES cohort revealed two genes that may be involved in PF pathogenesis, one of which is *TOM1L2*. Interestingly, in the study where Stuart et al. linked *PARN* and *RTEL1* to FPF through gene burden analysis, *TOM1L2* was among the top 10 genes of interest (based on *p*-value), although genome-wide significance was not reached [13]. We demonstrate here that TOM1L2 is expressed in the lung and in the AT2 cells, which are deemed the culprit cell in IPF. Information regarding the function of TOM1L2 is scarce. The variant results in an amino acid substitution in the highly conserved VHS domain. Normal telomere length in blood, and telomere length in AT2 cells that is comparable to sporadic IPF and longer than TERT-IPF, indicate a non-telomere-related pathway. Interestingly, *TOM1L2* RNA expression was increased in IPF lung compared to control, which suggests a role for TOM1L2 in disease, though it may be a response to disease processes.

TOM1L2, together with TOM1 and TOM1L1, forms a subfamily of VPS (Vps27-Hrs-STAM) containing proteins. TOM1L2 shows considerable overlap in amino acid sequence with TOM1 (59%) and TOM1L1 (30%) [36] In vitro a fragment of the TOM1L2 GAT domain binds TOLLIP [37] This is of specific interest, as the minor alleles of the common SNPs rs111521887 and rs5743894 in the *TOLLIP* gene are associated with IPF. These IPF risk alleles are associated with significantly reduced TOLLIP expression, whereas the minor allele of rs5743890 was found to be protective for IPF and associated with disease progression and worse survival [38,39]. In mice expressing low levels of TOM1L2, an abnormal immune response was observed which was characterized by increased incidence of skin and eye infections, splenomegaly, and tumors [40]. Possibly in line with the latter, in vitro, TOM1L2 overexpression exerts an inhibiting effect on mitogenesis [41]. This increased incidence of infections, combined with the well-known role of TOLLIP in the innate immune response with its role in IL1 and Toll-like receptor signaling, suggests a role for impaired host defense. However, our patients with the *TOM1L2* variant did not display a specifically inflammatory pulmonary phenotype.

On the other hand, mouse TOM1L2 is located in the trans-Golgi network and a role in vesicular transport and trafficking was suggested [40]. TOM1L2 also interacts with clathrin, and Katoh et al. hypothesize that—due to its similarity to TOM1 and TOM1L1—TOM1L2 is recruited by TOLLIP to endosomes and in turn recruits clathrin to endosomes [37]. TOM1L2 was further shown to interact in vitro with myosin VI by means of co-immunoprecipitation [42] and to bind polyubiquitin chains (both Lys48 and Lys63 linked chains) [43], pointing towards a role in endosomal sorting. Keeping in mind the significance of the AT2 cells in pulmonary fibrosis, it is interesting to speculate on a role for TOM1L2 in these cells. Several studies showed that mutations in *SFTPC* may result in abnormal trafficking via the plasma membrane followed by internalization to the endosomal compartment [44,45]. It was recently hypothesized that also wild type SFTPC may first travel to the plasma membrane [46]. It would subsequently be internalized via AP2-dependent endocytosis in a clathrin-coated vesicle [46], which was at least in enterocytes shown to depend on myosin VI [47], followed by cleavage in an early and then a later endosomal compartment. Lys63-chain ubiquitination is subsequently necessary for trafficking into the MVBs [46]. Some cautiousness is warranted as the majority of these experiments were performed in HeLa cells, rather than AT2 (resembling) cells [48]. However, it remains interesting that TOM1L2 was shown to associate with multiple players in this process.

The second gene we putatively linked with PF was *TDP1*. The overlapping variant caused a frameshift, resulting in a stop codon four amino acids downstream. We observed TDP1 protein expression in the lung and AT2 cells, as previously shown by Fam et al. [49]. Fam et al. further found that TDP1 is located mostly in cytoplasm of AT1 cells and more nuclear in AT2 cells. Additionally, they showed that during oxidative stress, TDP1 relocates to the mitochondria in cultured skin fibroblast. TDP1 is involved in different types of DNA repair, of both nuclear and mitochondrial DNA [50]. Here we studied whether presence of γH2AX is increased in AT2 cells of *TDP1* c.1373dupG variant carrier with IPF, but found fluorescence to be comparable to controls, in contrast with sporadic and TERT IPF samples where γH2AX was strongly increased. This was unexpected, as *TDP1*-knockdown human tumor cells that were treated with etoposide, which eventually causes DNA double strand breaks (DSBs), showed increased γH2AX levels [51]. Although we cannot link TDP1 function to pulmonary fibrosis, it is interesting to note that Kosmider et al. showed mitochondrial dysfunction in AT2 cells in emphysema, associated with increased mtDNA damage and reduced TDP1 levels. When tissue from areas with mild and severe emphysema from the same patient was compared, the TDP1 level was lower in the more affected areas [52]. Interestingly, emphysema was present in various degrees in three out of four *TDP1* variant carriers. The fourth carrier was diagnosed with LAM at age 46. HRCT showed limited thin-walled cysts, but no signs of emphysema or fibrosis were present on her most recent HRCT at age 45. However, the three other patients were diagnosed with disease at age 62, 50, and 56, thus increased awareness for signs of fibrosis may be warranted during clinical follow-up.

Genetic analysis in PF has shown that all dominant disease-causing variants are rare, and often unique for a specific family. This hampers detection of new genes and variants which has consequences for the diagnosis and management of patients. Clinical genetic analysis is a powerful tool and when a genetic cause can be identified, this aids with disease diagnosis and may provide information regarding prognosis, risk of comorbidities, and response to treatment. Additionally, it can be used to determine disease risk assessment in relatives and aid family planning [53]. This study shows that rare variant overlap analysis using an extensive biobank can be used to detect new genes and variants.

The study has several limitations. The strict variant filtering allowed only inclusion of previously unreported variants, increasing the risk of discarding shared pathogenic variants that are present at low frequency in the general population. Furthermore, only a small number of lung biopsies were available for analysis and as we did not perform functional experiments, the effect of the observed variants on protein function remains unknown. As there is considerable overlap between TOM1L2 and especially TOM1, it may be difficult to discriminate between the two in visualization of the protein. However, the rabbit anti-TOM1L2 antibody used here was also used by Protein Atlas (version 21.1 [54]). No TOM1L2 staining was reported there in alveolar cells, however we used a higher concentration (1:10 vs 1:75) and immunofluorescence staining, a more sensitive method.

The current study shows the potential of searching for overlapping variants as a tool to identify new putative disease-associated variants and with that, genes. In addition, it underlines the value of an extensive well-phenotyped cohort in rare disease. We detected two previously unreported variants, one in *TOM1L2* and one in *TDP1*, in multiple patients with pulmonary disease. To date, neither gene is related to telomere maintenance or the surfactant system, processes that currently cover all known PF genes. This suggests a new mechanism involved in PF disease pathogenesis and further research into the role of these genes and the observed variants is therefore needed.

## 4. Methods and Materials

### 4.1. Patients

We retrieved 1731 unrelated patients with PF from the St. Antonius ILD Center of Excellence Biobank (hereafter referred to as ‘ILD Biobank’) and first selected PF patients for whom a genetic variant was detected (n = 125) in established dominant genes associated with PF. These PF genes included *SFTPA2*, *SFTPC*, *ACD*, *PARN*, *POT1*, *RTEL1*, *TERC*, *TERT*, and *TINF2*.

Second, we selected a PF group for WES analysis based on presence of familial disease, age of presentation of ILD below 55 years, suspicion of short telomere syndrome or prior presence of whole exome data. WES was performed for 149 unrelated subjects with pulmonary fibrosis from the ILD Biobank. This includes 102 patients with pulmonary fibrosis in the context of familial disease (68.5%), 9 (6.0%) patients with sporadic pulmonary fibrosis were included because they were younger than 55 years old at diagnosis, 3 (2.0%) because there was suspicion of short telomere syndrome, and 35 (23.5%) IPF patients were previously sequenced as part of a study [4]. Variants of interest were screened using a TaqMan genotyping assay in a larger cohort of other patients with pulmonary fibrosis (n = 1583), non-PF pulmonary patients (n = 604 of which 85.8% had sarcoidosis) and healthy controls (n = 561), all registered in the ILD Biobank. Residual FFPE (lung) tissue from biopsy (Patients 2, 3, and 5) or autopsy (Patient 4) material was collected. The study was approved by the Medical Research Ethics Committees United (MEC-U) of the St. Antonius Hospital (approval number R05-08A) and subjects provided written informed consent.

### 4.2. Samples

The magnetic beads-based method (chemagic DNA blood 10k kit; Perkin Elmer Inc. Waltham, MA, USA) was used for genomic DNA extraction from peripheral leukocytes. Biopsy material was available from two patients with the *TOM1L2* variant and one with the *TDP1* variant. Biopsy material of control lung, sporadic IPF and *TERT* mutation carriers was included as a comparison. Autopsy material from one patient with the *TDP1* variant was available from lung, kidney, and liver tissue and compared to previously published data from healthy controls, sporadic IPF patients and an IPF patient carrying a *TERT* mutation [4].

### 4.3. Whole Exome Sequencing

Whole Exome Sequencing (WES) was performed in gDNA from peripheral leukocytes by Novogene (Hong Kong, China) using the Agilent Sure-Select Human All Exon V6 kit (Agilent Technologies, Santa Clara, CA, USA) on an Illumina PE150 sequencing platform (Illumina, San Diego, CA, USA) according to standard protocol. On average at least 20-fold read coverage was achieved for 93.7%. Reads were aligned to reference genome assembly hg19/GRCh37.

### 4.4. Variant Selection

Variant call files were uploaded into Ingenuity Variant Analysis (IVA) software (Qiagen, Hilden, Germany) for filtering. Variants were filtered to: (1) exclude variants present in population databases; (2) include variants that are deleterious as predicted by in silico prediction software; and (3) keep only variants that are present in at least two unrelated patients. For more detail, see Supplemental Methods S1 and Supplemental Results S1. Resulting monoallelic variants were subjected to visual inspection using the Integrative Genomics Viewer (IGV) tool (Supplemental Results S2).

### 4.5. Targeted Genetic Analysis

Sanger sequencing was used to check the *TOM1L2* c.421T > C, *TDP1* c.1373dupG and *TBXT* c.457A > C variants. For primers, see Supplemental Methods S2. *TOM1L2* c.421T > C and *TDP1* c.1373dupG were subsequently genotyped in an extended cohort using custom Taqman assays and the QuantStudio 5 Real-Time PCR system (both ThermoFisher Scientific, Waltham, MA, USA).

### 4.6. Immunofluorescent Staining of TOM1L2 and TDP1 in FFPE Diagnostic Lung Biopsies

Staining of TOM1L2 and TDP1 was based on a similar staining procedure as described previously [21]. In short, residual FFPE lung tissue from diagnostic biopsies were cut in 4 μm slices and stored at 4 °C until use. After antigen retrieval, slides were incubated with the following antibodies: mouse anti human TDP1 antibody (C-3, sc-365674, Santa Cruz, Dallas, TX, USA) previously used by Kosmider et al. [52], rabbit anti human TOM1L2 (1:10, HPA023304 R09917, Sigma-Aldrich, Saint Louis, MO, USA), and mouse anti human TOM1L2 (1:10, H00146691-B01 08071 WULZ, Abnova, Taipei, Taiwan). In case of the two mouse antibodies, slides were co-stained with an AT2-specific cell marker (rabbit anti-human pro-Spc antibody, 1:100, AB3786; Merck Millipore, Darmstadt, Germany) and corresponding immunofluorescence secondary antibodies. To reduce auto-fluorescent signals, slides were incubated with Vector TrueView (Vector laboratories, Newark, CA, USA) for 3 min and nuclei were subsequently counterstained with 4′,6-diamidino-2-phenylindole (DAPI; 25 μg/mL). Pictures were taken with a LSM700 laser scanning confocal microscope (Zeiss, Jena, Germany).

### 4.7. Measurement of Average Telomere Length in Tissue and Blood Using MMqPCR

Slices of FFPE tissue from lung were deparaffinated using paraffin dissolver (Macherey-Nagel, Düren, Germany). DNA was isolated using an AllPrep DNA/RNA FFPE Kit (Qiagen, Hilden, Germany) and quantified using Nanodrop (Thermo Fisher Scientific, Waltham, MA, USA). Measurement of telomere length in peripheral blood leukocytes and FFPE tissue from autopsy section material was performed as previously described (Van Batenburg et al., 2020). To determine T/S ratios as a measure for average relative telomere length, monochrome multiplex qPCR (MMqPCR) was performed on a CFX96™ Single-Color Real-Time PCRDetection System (Bio-Rad, Hercules, CA, USA) using iQ SYBR Green Supermix (Bio-Rad, Hercules, CA, USA).

### 4.8. Quantification of TOM1L2 Expression with qPCR

The expression of *TOM1L2* RNA was measured in lung tissue obtained from biopsy. RNA was isolated from formalin-fixed paraffin-embedded tissue using AllPrep DNA/RNA FFPE Kit (Qiagen, Hilden, Germany). Paraffin was removed first using paraffin dissolver (Machery-Nagel, Düren, Germany). RNA concentration and purity was measured by nanodrop and RNA was reversed transcribed using iScript (Bio-Rad, Hercules, CA, USA). 6 ng cDNA was used in real time RT-PCR which was performed on a CFX96 Single-Color Real-Time PCRDetection System (Bio-Rad, Hercules, CA, USA) using iQ SYBR Green Supermix (Bio-Rad, Hercules, CA, USA). The quantitation was made with the comparative threshold cycles (delta Ct*1000) method where the amount of *TOM1L2* target was normalized to the mean of 3 endogenous reference genes: *RPL13A*, *beta-actin* and *EEF1A1* see Supplemental Methods S2 for primers. Measurements were performed in duplicate.

### 4.9. Statistical Analysis

Statistical analyses were performed using GraphPad Prism (version 8.3). Differences between groups were analyzed by Mann–Whitney U tests or, in case of multiple groups, Kruskal–Wallis tests as appropriate for non-parametric data. The latter was followed by Dunn’s multiple comparisons test in case of a significant outcome. Values of *p* < 0.05 were considered as statistically significant.

## Figures and Tables

**Table 1:** Overlapping variants in established pulmonary fibrosis (PF) genes

Gene	Number of Unrelated Patients	Transcript	Nucleotide Change	Amino Acid Change	ACMG Classification	Publication *
*PARN*	2	NM_001134477.2	c.1214G > C	p.(Ser405Thr)	VUS	
*PARN*	4	NM_001134477.2	c.98C > T	p.(Pro33Leu)	VUS	Dressen et al. (2018); Justet et al. (2021); Van Batenburg et al. (2018) [21,22,23]
*POT1*	2	NM_015450.2	c.776T > C	p.(Leu259Ser)	LP	Kelich et al. (2022) [20]
*RTEL1*	3	NM_001283009.1	c.1972_1974del	p.(Leu658del)	LP	Justet et al. (2021) [22]
*RTEL1*	2	NM_001283009.1	c.3811C > T	p.(Arg1271Trp)	VUS	
*RTEL1*	5	NM_001283009.1	c.2956C > T	p.(Arg986 *)	P	Borie et al. (2019); Moriya et al. (2016); Van Batenburg et al. (2020) [4,24,25]
*SFTPA2*	2	NM_001098668.2	c.629A > C	p.(Asn210Thr)	LP	Van Moorsel et al. (2015) [26]
*SFTPC*	3	NM_003018.3	c.218T > C	p.(Ile73Thr)	P	Nogee et al. (2002); Van Moorsel et al. (2010) and many others [27,28]
*TERT*	2	NM_198253.2	c.2701C > T	p.(Arg901Trp)	P	
*TERT*	2	NM_198253.2	c.1726C > T	p.(Tyr576His)	VUS	
*TERT*	3	NM_198253.2	c.1584T > G	p.(Cys528Trp)	LP	Justet et al. (2021) [22]
*TERT*	4	NM_198253.2	c.2312C > T	p.(Pro771Leu)	LP	
*TERT*	2	NM_198253.2	c.2594G > A	p.(Arg865His)	P	Diaz de Leon et al. (2010); Justet et al. (2021); Newton et al. (2016); Tsakiri et al. (2007) [5,22,29,30]
*TERT*	2	NM_198253.2	c.299G > A	p.(Gly100Asp)	VUS	Justet et al. (2021) [22]
*TERT*	13	NM_198253.2	c.2005C > T	p.(Arg669Trp)	LP	Dressen et al. (2018); Justet et al. (2021); Newton et al. (2016); van der Vis et al. (2020) ^#^ [5,19,22,23]

VUS = variant of unknown significance; LP = likely pathogenic; P = pathogenic variant as classified according to the ACMG guidelines [31]. ^#^ post-hoc genealogical analysis revealed distant relatedness to the fourth degree and up, see references for details), * = Publications in italics are reports including patients from the Dutch PF cohort presented in this paper.

**Table 2:** Variants of interest. TDP1 and TOM1L2 are located on chromosomes 14 and 17, respectively. Predictions by SIFT [32], PolyPhen-2 [33], and CADD score were obtained from the IVA software. Prediction by Mutation Taster was obtained from Mutation Taster2021 [34].

Gene	Transcript	Nucleotide Change	Amino Acid Change	Translation Impact	SIFT	PolyPhen-2	CADD	Mutation Taster
*TDP1*	NM_018319.4	c.1373dupG	p.(Ser459fs*5)	frameshift	-	-	35.0	Deleterious
*TOM1L2*	NM_001288786.2	c.421T > C	p.(Tyr141His)	missense	Damaging	Probably Damaging	28.1	Benign (deleterious for all other transcipts)

**Table 3:** Clinical characteristics of *TOM1L2* c.421T > C or *TDP1* c.1373dupG variant carriers. fHP = fibrotic hypersensitivity pneumonitis; IPF = idiopathic pulmonary fibrosis; LAM= lymphangioleiomyomatosis; LTx = lung transplant; b = bilateral and u = unilateral transplant; UIP = usual interstitial pneumonia; NSIP = nonspecific interstitial pneumonia; DAD = diffuse alveolar damage; DIP = desquamative interstitial pneumonia; TL = telomere length in peripheral blood. ^#^ = biopsy tissue was used in this study.

ID	Variant	Familial Disease	Sex	Diagnosis	Age at Diagnosis (Death)	Age LTx	HRCT Pattern	Histology (Tissue)	TL < 10th Percentile for Age
Patient 1	*TOM1L2* c.421T > C	Yes	Male	fHP + secondary UIP characteristics	52	60 (b)	probable UIP	UIP characteristics (explant)	No
Patient 2 ^#^	*TOM1L2* c.421T > C	No	Male	IPF	47 (55)	51 (u)	UIP	UIP + DIP component (explant)	Yes
Patient 3 ^#^	*TOM1L2* c.421T > C	No	Male	NCIP	55 (59)	NA	NSIP	UIP (VATS biopsy)	No
Patient 4	*TDP1* c.1373dupG	Yes	Male	IPF	62 (63)	NA	UIP with DAD	UIP (VATS biopsy)	Yes
Patient 5 ^#^	*TDP1* c.1373dupG	No	Male	IPF	50 (61)	52 (b)	UIP, paraseptal emphysema	UIP (explant)	No
Patient 6	*TDP1* c.1373dupG	No	Female	LAM	46	NA	multiple thin-walled cysts	NA	No
Patient 7	*TDP1* c.1373dupG	No	Male	pretransplant COPD/emphysema; posttransplant progressive pulmonary fibrosis in right lung with UIP characteristics	56 (62)	57 (u)	emphysema, UIP	emphysema, presence of fibroblast foci (explant)	Yes

## Data Availability

Data can be requested from the corresponding author.

## Supplementary Materials

The supporting information can be downloaded at: https://www.mdpi.com/article/10.3390/ijms24032790/s1.
