# Supplementary material for: Genetic Variant Overlap Analysis Identifies Established and Putative Genes Involved in Pulmonary Fibrosis
Source: Int J Mol Sci. 2023 Feb 1;24(3):2790. doi: 10.3390/ijms24032790 (PMC9917193; doi:10.3390/ijms24032790)
Supplement: Supplementary file 1 [file ijms-24-02790-s001.zip › ijms-2132947-supplementary.pdf]

SUPPLEMENT

Supplemental Methods

Supplemental Methods S1. Variant filtering in IVA

Variant call files were uploaded into Ingenuity Variant Analysis (IVA; Qiagen) software for filtering.
Confidence filter: Only variants were kept that passed upstream pipeline filtering and with 1) call quality of ≥20; 2) read depth ≥10; 3) genotype quality ≥30; allele fraction ≥25; 4) outside top 5% most exonically variable 100base windows in healthy public genomes; 5) outside top 1% most exonically variable genes in healthy public genomes (1000 genomes).
Common variants filter: to exclude known variants, variants with frequency ≥0.001 in either 1000 Genomes, or the NHLBI ESP populations or ≥0.001 in the subpopulations with the highest allele frequency of the ExAC or gnomAD databases, were excluded, as well as variants present in dbSNP.
Predicted deleterious filter: Only variants no more than 20 bases into an intron were kept. Variants were included if they are associated with loss of function of a gene by causing a 1) frameshift, in-frame indel, or start/stop codon change; or 2) are a missense variant unless predicted tolerated by SIFT or PolyPhen-2; or 3) Predicted deleterious by having CADD score >25; or 4) Splice site loss up to 2 bases into intron or as predicted by MaxEntScan; or 5) Copy Number Loss. Subsequently, variants are only kept if they are pathogenic, likely pathogenic or of uncertain significance according to ACMG classification guidelines as computed by the IVA software. Any remaining variants with a Combined Annotation-Dependent Depletion (CADD v1.6) score <20 were excluded. CADD integrates various different annotations into one Phred-based score of deleteriousness for SNVs and indels[54].
Genetic filtering: Exclude variants homoyzgous in ≥ 1 sample and subsequently, keep only variants present in ≥ 2 samples.

Supplemental Methods S2. Primers

Primers used for targeted genetic analysis

**Name**	**forward primer (5'-3')**	**reverse primer (5'-3')**	**product length**
TOM1L2	CTCCTGCATGCCCCTTGTTA	GTTGCGGAAGGAGAGGAACA	456
TBXT exon 3	AGAGAAGGCTGTGGCAGTTT	TGCCCCGAAATAACTGAGGC	499
TDP1 exon 13	CCTCTTAATGAGGGATCCTC	GTGACATAAATAACACTCTAATTCC	195

qPCR primers used for quantification of TOM1L2 expression

**Name**	**forward primer (5'-3')**	**reverse primer (5'-3')**	**product length**
TOM1L2	CAGGCTGCGGAGTGAACTG	TCCAGGGACCATTTCTGTTAACA	78
ACTB	CATTCCAAATATGAGATGCGTTGT	TGTGGACTTGGGAGAGGACT	103
RPL13A	CCTGGAGGAGAAGAGGAAAGAGA	TTGAGGACCTCTGTGTATTTGTCAA	126
EEF1A1	CATCAAAGCAGTGGACAAGAAG	GGGTGGCAGGTATTAGGGATAA	104

Supplemental Results

Supplemental Results S1. Flow chart of the variant filtering steps

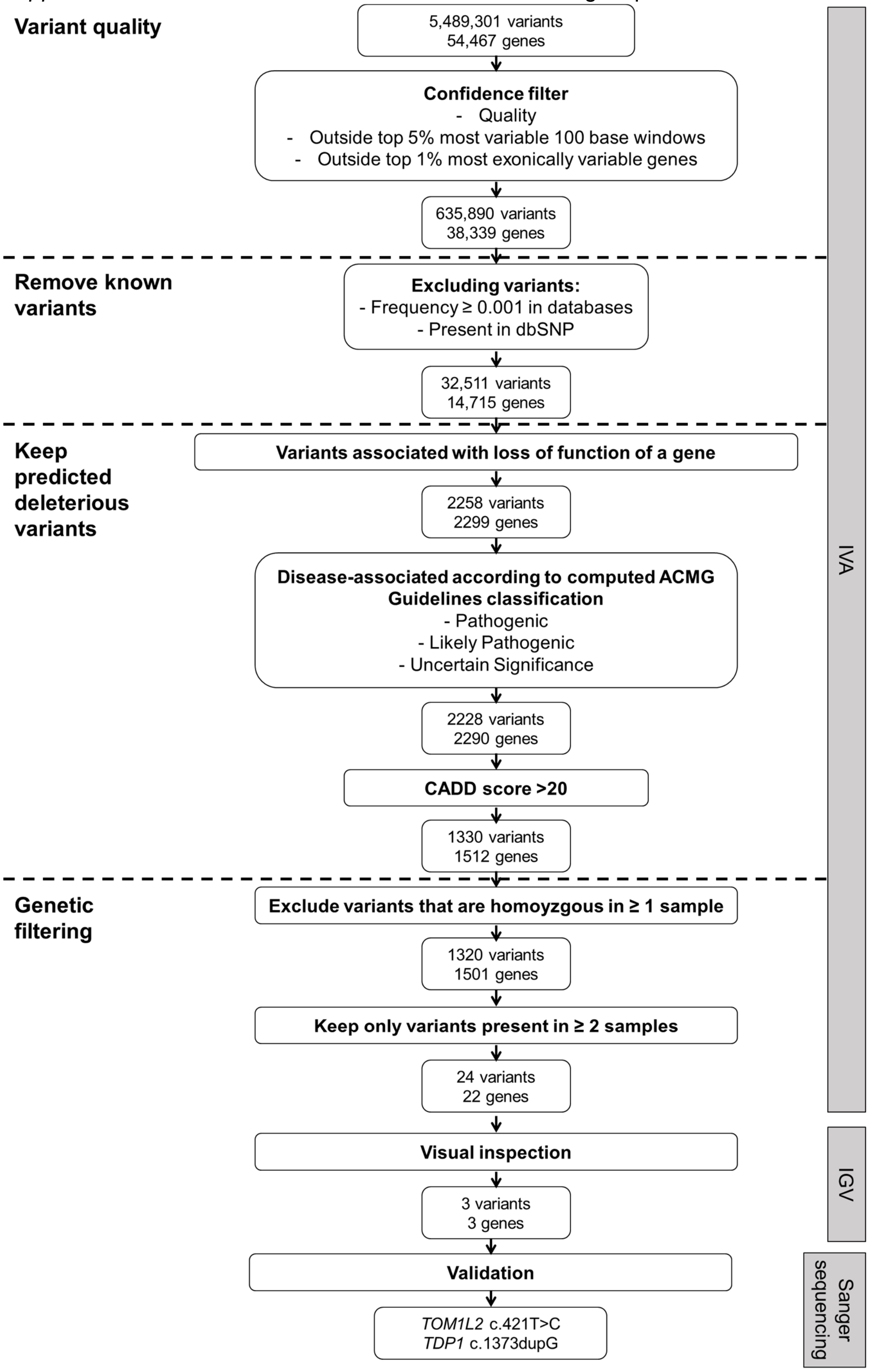

Flow chart of the variant filtering steps. IVA = Ingenuity Variant Analysis; IGV = Integrative Genomics Viewer

Supplemental Results S2. Excluded variants

Gene	Transcript	Nucleotide change	Amino acid change	CADD	SIFT*	PolyPhen-2[#]	No. variant carriers
ANKRD36C	ENST00000456556.5	c.4529_4530insA	p.(L1511fs*9)	23.400	-	-	6
ANKRD36C	ENST00000456556.5	c.4531dupC	p.(L1511fs*9)	24.800	-	-	6
ARID5B	ENST00000279873.12	c.1005G>T	p.(R335S)	25.000	D	ProD	2
FBXW2	ENST00000608872.6	c.683C>G	p.(A228G)	34.000	T	ProD	4
FBXW2	ENST00000608872.6	c.677C>G	p.(T226R)	28.000	T	ProD	3
FRG1BP	ENST00000278882.8	n.417+1G>C	-	22.400	-	-	6
KIR2DL4	ENST00000345540.9	c.676_677insAT	p.(P226fs*15)	20.800	-	-	5
KIR2DL4	ENST00000345540.9	c.674_675delGG	p.(W225fs*5)	23.300			5
KRT77	ENST00000341809.8	c.1292_1293insCACTACAAACTCC GCCTCCTGGGTCCAAGTGATTCT CCTGCCTCAGCCTCCTGAGTAGCTG	p.(E432fs*19)	25.600	-	-	3
LRR1	ENST00000298288.11	c.1049A>G	p.(D350G)	24.500	D	ProD	2
MED13	ENST00000397786.7	c.6269A>G	p.(Y2090C)	25.700	D	PosD	2
NUP210L	ENST00000368559.7	c.1612G>T	p.(E538*)	36.000	-	-	2
OR5AP2	ENST00000544374.2	c.478G>T	p.(G160*)	35.000	-	-	2
PRKRA & PJVK	ENST00000325748.9	PRKRA: c.66-4_66-3insTCCCTTC TCGCCCTGTCCCAGAGCAGGC ACCGCCGAGGCCCCGCCGCT GGAGCGCGAGGACAGTGGGA CCTT	-	20.500	-	-	3
	ENST00000375129.8	PJVK: c.-1378_-1377insAAGGTC CCACTGTCCTCGCGCTCCAGC GGCGGGGCCTCGGCGGTGCC TGCTCTGGGACAGGGCGAGA AGGGA					
RUNX1	ENST00000300305.7	c.1270T>G	p.(S424A)	25.800	T	B	2
SLC29A2	ENST00000357440.7	c.1258A>C	p.(R420R)	23.400	-	-	2
TBXT	ENST00000296946.6	c.457A>C	p.(N153H)	27.500	D	PosD	3
TPSD1	ENST00000335725.9	c.521-2A>C	-	22.700	-	-	2
USF3	ENST00000316407.9	c.4416_4418delGCA	p.(Q1478del)	21.800	-	-	42
USF3	ENST00000316407.9	c.4413_4418dupGCAGCA	p.(Q1477_Q1478dup)	21.300	-	-	5
USF3	ENST00000316407.9	.4416_4418dupGCA	p.(Q1478dup)	21.400	-	-	4
USF3	ENST00000316407.9	c.4416_4418delGCA	p.(Q1478del)	20.500	-	-	2

Variants present in at least 2 patients identified using IVA software. None of the variants is found in dbSNP or gnomAD. CADD score, SIFT, and PolyPhen-2 predictions are provided by the IVA software. *D=Damaging; T=Tolerated; [#]B=Benign; PosD=Possibly damaging; ProD=Probably damaging

Variant	Reason for exclusion
*ANKRD36C c.*4529_4530insA p.(L1511fs*9)	variant is not convincing based on visual inspection of raw data in IGV
ANKRD36C c.4531dupC p.(L1511fs*9)	variant is not convincing based on visual inspection of raw data in IGV
ARID5B c.1005G>T p.(R335S)	variant is not convincing based on visual inspection of raw data in IGV

FBXW2 c.683C>G p.(A228G)	variant is not convincing based on visual inspection of raw data in IGV
FBXW2 c.677C>G p.(T226R)	variant is not convincing based on visual inspection of raw data in IGV
FRG1BP n.417+1G>C	variant is not convincing based on visual inspection of raw data in IGV
KIR2DL4 c.676_677insAT p.(P226fs*15)	variant is not convincing based on visual inspection of raw data in IGV
KIR2DL4 c.674_675delGG p.(W225fs*5)	variant is not convincing based on visual inspection of raw data in IGV
KRT77 c.1292_1293insCAC… p.(E432fs*19)	variant is not convincing based on visual inspection of raw data in IGV
LRR1 c.1049A>G p.(D350G)	for each carrier WES data of one affected relatieve was available. Variant does not segregate in either family
MED13 c.6269A>G p.(Y2090C)	a pathogenic variant in another PF gene was identified in one carrier. As only one variant carrier thus remained, the variant no longer fullfilled the selection criteria.
NUP210L c.1612G>T p.(E538*)	variant is not convincing based on visual inspection of raw data in IGV
OR5AP2 c.478G>T p.(G160*)	both carriers additionally carry a c.479G>T variant *in cis*. The result of these combined variants is an amino acid alteration (predicted 'benign' by PolyPhen) instead of a premature stop codon
PRKRA & *PJVK*	variant is not convincing based on visual inspection of raw data in IGV
RUNX1 c.1270T>G p.(S424A)	variant is not convincing based on visual inspection of raw data in IGV
SLC29A2 c.1258A>C p.(R420R)	variant is not convincing based on visual inspection of raw data in IGV
TBXT c.457A>C p.(N153H)	not confirmed by Sanger sequencing
TPSD1 c.521-2A>C	variant is not convincing based on visual inspection of raw data in IGV
*USF3 c.*4416_4418delGCA p.(Q1478del)	variant is not convincing based on visual inspection of raw data in IGV
USF3 c.4413_4418dupGCAGCA p.(Q1477_Q1478dup)	variant is not convincing based on visual inspection of raw data in IGV
USF3 c.4416_4418dupGCA p.(Q1478dup)	variant is not convincing based on visual inspection of raw data in IGV
USF3 c.4416_4418delGCA p.(Q1478del)	variant is not convincing based on visual inspection of raw data in IGV

Reason for exclusion of variants which passed the initial IVA filters. IGV = Integrative Genomics Viewer

Supplemental Results S3. TOM1L2 immunofluorescence using rabbit-anti-TOM1L2 primary antibody

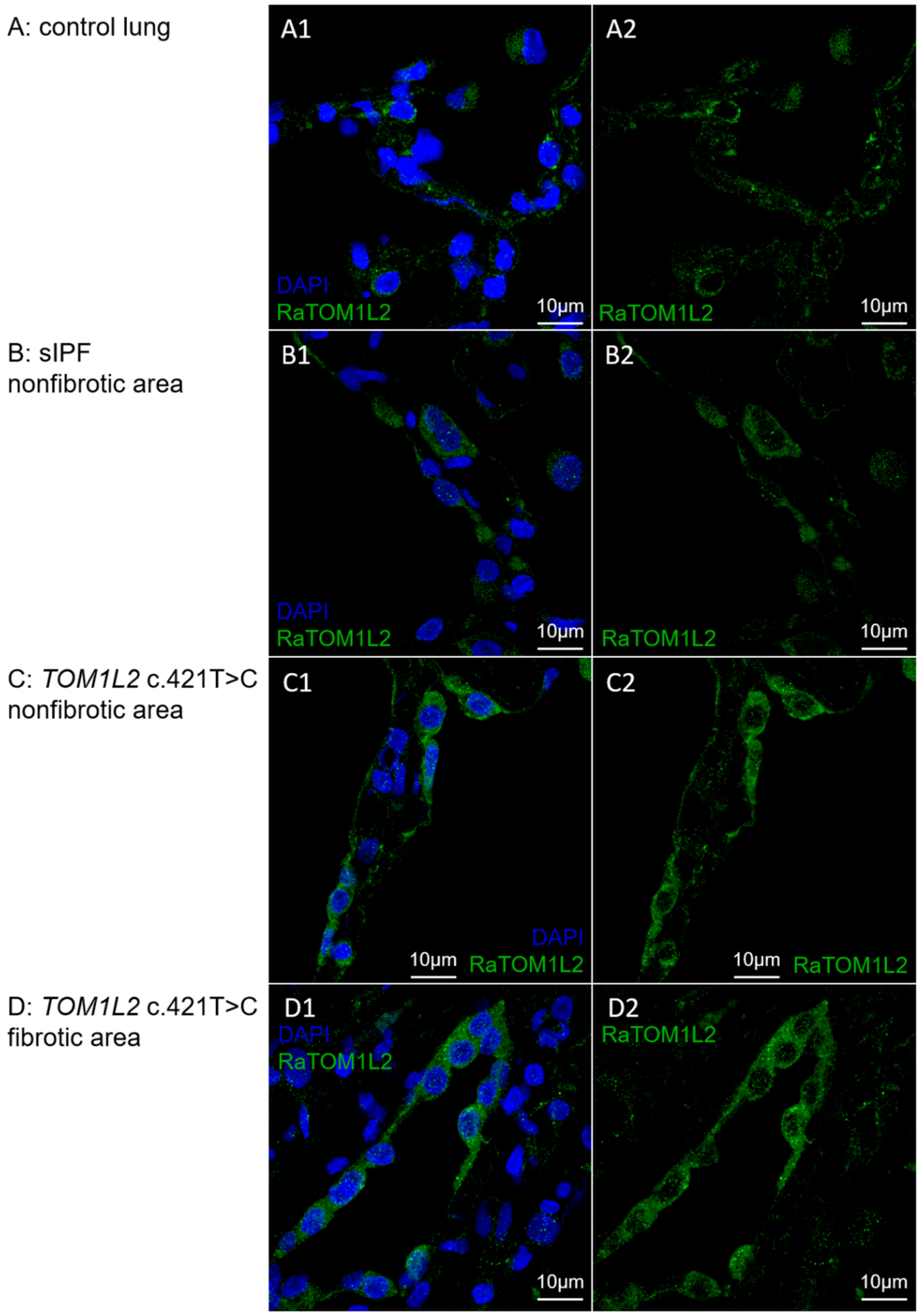

TOM1L2 immunofluorescence in lung tissue of control subject (A), non-fibrotic area in sporadic IPF lung (B), non-fibrotic area of a *TOM1L2* c.421T>C variant carrier (C) and in fibrotic area of a *TOM1L2* c.421T>C variant carrier. A2-D2 show staining with rabbit-anti-TOM1L2 antibody and A1-D1 show co-staining of rabbit-anti-TOM1L2 and DAPI.

Supplemental Results S4. TOM1L2 RNA expression in whole lung

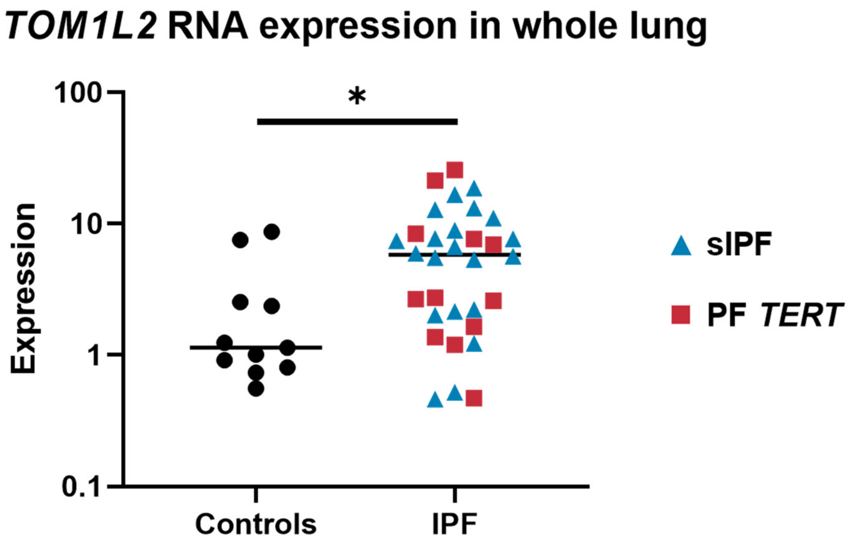

TOM1L2 RNA expression in whole lung from controls and IPF patients. Blue triangles: sporadic IPF patients (sIPF); red squares: IPF patients with a *TERT* mutation (TERT). *TOM1L2* expression is relative compared to the mean of 3 reference genes multiplied by 1000. * = p<0.05, calculated by Mann Whitney test.
